# Advance Nanomaterials for Biosensors

**DOI:** 10.3390/bios12040219

**Published:** 2022-04-07

**Authors:** Sadanand Pandey

**Affiliations:** Department of Chemistry, College of Natural Science, Yeungnam University, 280 Daehak-Ro, Gyeongsan 38541, Korea; sadanand.au@gmail.com or spandey@ynu.ac.kr

Nanotechnology has a significant impact on everything in our daily life. Nanomaterial-enabled sensors are being designed for highly sensitive and selective, fast response, inexpensive, and large-scale production with great reliability, multiplex-functionality, and high-flexibility sensing applications. The demand for the production of rapid sensors for a wide range of applications, namely health diagnostics, medical engineering, environmental analysis, food safety/quality control, and detection of toxic metabolites, is of increasing interest for researchers the world over. The use of nanomaterials in biosensors is very promising because they mediate current flow. Surface modification of the electrodes, based on various novel nanomaterials (such as carbon nanomaterials, metal nanoparticles, nanofibers, nanowires, and nanotubes, etc.), significantly increases the performance of the biosensors. Chemical stability, high current density, and complex surface chemistry result in desirable properties in nanomaterials for developing such sensors. Ultimately, this implementation will enhance the sensor sensitivity and stability. The present Special Issue aims at presenting new research work on advanced nanomaterial-based biosensors, particularly in the areas of new approaches to synthesizing, characterizing, and modifying nanomaterials for detecting analytes of interest in environmental and medical sciences. This Special Issue also summarizes the most recent findings and future challenges regarding biosensors.

In this collection, we combined five outstanding contributions focusing on different aspects of the biosensing field, mostly highlighting the designing of nanomaterial-based biosensors for diagnosing and treating cancers such as retinoblastoma and osteosarcoma. It also highlights the practical design of nanoscale devices to detect alkaline phosphatase quantitatively in clinical diagnosis. The various progress in the area of non-enzymatic sensing of dual/multi biomolecules, developments in non-enzymatic glucose and H_2_O_2_ (NEGH) sensing, multi-functionalized nanocarrier therapies for targeting retinoblastoma, galactose functionalized nanocarriers, sensing performance, electrocatalytic mechanism, morphology and design of electrode materials are also thoroughly reviewed. The biosensors, along with their applications and the benefits of machine learning, innovative approaches to improve the NEGH sensitivity, selectivity and stability in real-time applications, and challenges and solutions in the field of biosensors, are also a major highlight of this Special Issue. A brief summary of each accepted contribution is provided below to encourage the readers to go through them and “visualize” the state of the art within the field of biosensing.

The most common type of metastatic cancer of the bones is osteosarcoma, which largely affects children and young adults, but can attack older adults as well [1]. Nanotechnology offers groundbreaking solutions for diagnosing and treating osteosarcoma through its applications in the clinical setting [2]. In a review published by Barani et al. [3], the researchers discussed possible applications of engineered nanomaterials in osteosarcoma diagnosis and treatment, which motivated them to develop new approaches to deal with the challenges associated with it. The authors conclude by suggesting that some nano polymeric materials are not very strongly cytotoxic, so it is likely that they will be offered to humans in the coming years. Nanotechnology will therefore play a crucial role in osteosarcoma diagnosis in the future. In the future, more powerful diagnostic techniques such as multimodal imaging will be available with the development of technology.

Apart from osteosarcoma, retinoblastoma is also one of the rare types of cancer, and its treatment, as well as diagnosis, is challenging, owing to mutations in the tumor-suppressor genes and lack of targeted, efficient, cost-effective therapy, exhibiting a significant need for novel approaches to address these concerns. Arshad et al. [4] examined recent advancements and potential development areas in the realm of intraocular medication delivery and diagnostic platforms using nanotechnology. In this review article, the authors have highlighted and reviewed various nanoparticles, muti-functionalized nanocarriers therapies which include Surface-Modified Melphalan Nanoparticles for the Intravitreal Chemotherapy of RB, Galactose Functionalized Nanocarriers, Hyaluronic Acid (HA) Functionalized Nanocarriers, Folic Acid (FA) Functionalized Nanocarriers, etc. The review paper [4] focuses on numerous diagnostic and therapeutic strategies that use diverse nanomaterials. The authors concluded that the barriers in the treatment of retinoblastoma and the killing of healthy cells have been minimized via using biocompatible polymers such as ligands and green synthesis-based metallic NPs, as well as bioactive nontoxic herbal flavonoid constituent-based lipid nanoparticles. Emerging trends of multi-functionalization and biocompatible ligands in anticancer therapy and diagnosis are opening a new era in overcoming the barriers of conventional therapies via strategically improving the treatment and diagnosis of retinoblastoma.

We currently know that non-enzymatic sensing has been a hot topic in research, and most nanomaterial-based sensors are designed to detect single analytes. The development of sensing elements for detecting glucose and hydrogen peroxide (H_2_O_2_) is important in this regard. Non-enzymatic sensing is more economical and has a longer lifetime than enzymatic electrochemical sensing, but it has several drawbacks, such as high working potential, slow electrode kinetics, poisoning from intermediate species and weak sensing parameters. Thatikayala et al. [5] comprehensively review the recent developments in non-enzymatic glucose and H_2_O_2_ (NEGH) sensing by focusing mainly on the sensing performance, electro catalytic mechanism, morphology and design of electrode materials. Various types of nanomaterials with metal/metal oxides and hybrid metallic nanocomposites are discussed. A comparison of glucose and H_2_O_2_ sensing parameters using the same electrode materials is outlined by Thatikayala et al. [5] in order to predict the efficient sensing performance of advanced nanomaterials. Recent innovative approaches to improve the NEGH sensitivity, selectivity and stability in real-time applications are critically discussed by authors, which have not been sufficiently addressed in the previous reviews. Finally, the challenges, future trends, and prospects associated with advanced nanomaterials for NEGH sensing are considered.

As an important DNA 3′-phosphatase, alkaline phosphatase can repair damaged DNA caused by replication and recombination. It is essential to measure the level of alkaline phosphatase to indicate some potential diseases, such as cancer, related to alkaline phosphatase. Wang et al. [6] designed a simple and fast method to detect alkaline phosphatase quantitively. When alkaline phosphatase is present, the resulting poly T-DNA with a 3′-hydroxyl end was cleaved by exonuclease I, prohibiting the formation of fluorescent copper nanoparticles. However, the fluorescent copper nanoparticles can be monitored with the absence of alkaline phosphatase. Wang et al. [6] can detect alkaline phosphatase with this turn-off strategy. The proposed method by Wang et al. is able to quantify the concentration of alkaline phosphatase with the LOD of 0.0098 U/L.

Finally, in the last review article, the authors focus on electrochemical biosensors. Electrochemical biosensors depict propitious diagnostic technology, which can detect biomarkers in body fluids such as sweat, blood, feces, or urine. Singh et al. [7] review the recent advances in electrochemical biosensors where they have highlighted various machine-learning tools and techniques that are used for biosensing are discussed briefly along with their applications and limitations such as Catalytic Biosensors, Affinity Biosensors, etc. The various electrochemical biosensors that have been developed are reviewed in detail by Singh et al. [7]. Presently, electrochemical biosensors are helping in combining biology with electronics. The biosensors are becoming efficient, smaller, and cost-effective. In the future, the electrochemical biosensor will revolutionize the field of diagnosis, health care, food security, and defense.
